# Simultaneous quantification of drug and coformer during cocrystal dissolution using *in situ* UV spectroscopy and multicomponent analysis

**DOI:** 10.5599/admet.3274

**Published:** 2026-04-17

**Authors:** Shiori Ishida, Samuel Lee, Balint Sinko, Karl Box, Kiyohiko Sugano

**Affiliations:** 1 Molecular Pharmaceutics Lab., College of Pharmaceutical Sciences, Ritsumeikan University, 1-1-1, Noji-higashi, Kusatsu, Shiga 525-8577, Japan; 2 Pion Inc. (UK) Ltd. Forest Row Business Park, Station Road, East Sussex, RH18 5DW, United Kingdom

**Keywords:** Carbamazepine, congruent, supersaturation, precipitation inhibitor, *in situ* UV probe

## Abstract

**Background and purpose:**

The primary objective of this study was to assess the feasibility of applying *in situ* UV spectroscopy in combination with multicomponent analysis (MCA) to simultaneously quantify the dissolution of a drug and its coformer from a cocrystal. A secondary objective was to determine whether this approach can support a mechanistic understanding of cocrystal dissolution.

**Experimental approach:**

The rotating-vessel μDISS system equipped with an *in situ* UV probe was used for dissolution tests. Carbamazepine (CBZ) cocrystals with saccharin, nicotinamide, and 2,4-dihydroxybenzoic acid were used as model compounds. The concentrations of CBZ and each coformer were simultaneously quantified using MCA of the *in situ* UV spectra.

**Key results:**

The concentrations of CBZ and the coformer were successfully quantified by MCA throughout the dissolution process. In the absence of a polymeric precipitation inhibitor (PPI), the dissolution of CBZ from the cocrystal reached only 20%, and no supersaturation was observed, whereas the coformers were rapidly released. In contrast, in the presence of PPIs, the dissolution of CBZ from the cocrystal increased to supersaturated levels, while the dissolution of the coformer decreased. Supersaturation of CBZ was achieved when CBZ and the coformer dissolved congruently. A PPI may interfere with the molecular dissociation of CBZ and the coformer from the cocrystal surface, resulting in a slower release of both CBZ and the coformer. This effect may have reduced the local CBZ concentration at the particle surface and, consequently, slowed the precipitation of CBZ dihydrate on the particle surface.

**Conclusion:**

MCA enables the simultaneous quantification of a drug and its coformer from *in situ* UV spectra during cocrystal dissolution testing. This analytical approach provides valuable insights into the dissolution mechanisms of cocrystals.

## Introduction

Recently, many drug candidates have been reported to exhibit poor solubility in aqueous media [1]. To address this issue, drug candidates can be formulated as cocrystals [2,3]. Previous studies have shown that drug cocrystals can exhibit a wide range of dissolution behaviours depending on the combination of coformers and polymeric precipitation inhibitors (PPI) [4-13]. A mechanistic understanding of cocrystal dissolution is critical for efficient coformer screening and rational formulation design.

In drug discovery and development, small-scale dissolution tests equipped with an *in situ* UV probe have been used for the screening of active pharmaceutical ingredients and formulations [14]. In most cases, attention has been focused solely on the dissolution of the drug. However, simultaneous determination of the drug and the coformer may provide deeper insight into cocrystal dissolution [15]. Theoretically, multicomponent analysis (MCA) enables decomposition of the UV spectrum of a sample that contains multiple components based on the standard UV spectra of each component [16-18]. The method is based on a modified classical least-squares technique to determine the contribution of each standard spectrum to the superposition by minimizing the difference (for details, see the supplemental information). However, its applicability to cocrystal dissolution has not been investigated.

**Figure 1.**
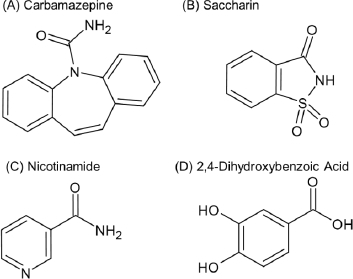
Chemical structures of drugs and coformers used in this study

The primary objective of this study was to evaluate the feasibility of applying *in situ* UV spectroscopy in combination with MCA to simultaneously quantify the dissolution of a drug and its coformer from a cocrystal. A secondary objective was to determine whether this approach can support a mechanistic understanding of cocrystal dissolution. Carbamazepine (CBZ) cocrystals of saccharin (SAC), nicotinamide (NIC) and 2,4-dihydroxybenzoic acid (DHBA) were used as model compounds (Figure 1). Polyethylene glycol 6000 (PEG), polyvinylpyrrolidone K30 (PVP), hydroxypropyl methylcellulose (HPMC), hydroxypropyl methylcellulose acetate succinate (HPMC-AS), and amino methacrylate copolymer (EPO) were employed as PPIs. The μDISS Profiler™ system (Pion Inc., Billerica, USA) equipped with an *in situ* UV probe was used for the dissolution test. Since the use of a magnetic stirrer can enhance drug precipitation via contact-induced nucleation [19], the rotating vessel (RV) method was used for agitation in this study [20].

## Experimental

### Material

Carbamazepine anhydrous form (CBZ AH), saccharin (SAC), nicotinamide (NIC), 2,4-dihydroxybenzoic acid (DHBA), sodium dihydrogen phosphate dihydrate, sodium chloride, 8 M NaOH, polyvinylpyrrolidone K30 (PVP), polyethylene glycol 6000 (PEG), and dimethylacetamide (DMA) were purchased from FUJIFILM Wako Pure Chemical Corporation (Osaka, Japan). Hydroxypropyl methylcellulose (Hypromellose TC-5®) (HPMC) and hydroxypropyl methylcellulose acetate succinate (Shin-Etsu AQOAT® MF) (HPMC-AS) were obtained from Shin-Etsu Chemical Co., Ltd (Tokyo, Japan). Amino methacrylate copolymer (Eudragit® EPO) (EPO) was obtained from Evonik Japan Co., Ltd (Tokyo, Japan).

CBZ-SAC (1:1) and CBZ-NIC (1:1) cocrystals and carbamazepine dihydrate (CBZ-DH) were prepared as previously reported [4]. CBZ-DHBA (1:1) was prepared by recrystallization from methanol (equimolar mixture of CBZ and DHBA) [21]. The cocrystal particles were sieved using a 125-μm (on) and 250-μm (pass) meshes before use for the dissolution tests.

### Methods

The μDISS Profiler™ system (Pion Inc., Billerica, USA) was used to conduct the dissolution tests. The details of the rotating vessel (RV-μDISS) method have been reported previously [20]. A small paddle made of a polyoxymethylene plate (20 mm, approximately 1/4 of the conventional paddle) was attached to the UV probe. The vessel was rotated at 50 rpm. Dissolution tests were performed at 37.0 ± 0.5 °C. A phosphate buffer solution (pH 6.5, 8 mM; ionic strength (*I*) = 0.14 M, adjusted with NaCl) was used as the dissolution medium. Each PPI was added at 0.1 wt.%. The test medium (15 mL) was added to each vessel, followed by the addition of each cocrystal (0.062 mmol; 4.2 mM), reflecting the clinical dose-to-intestinal fluid volume ratio (200 mg dose as of CBZ in the fluid volume of 50-250 mL). The UV spectra were continuously monitored using the *in situ* UV probe with 1 mm apertures. The drug and coformer concentrations (*C*_CBZ_, *C*_SAC_, *C*_NIC_, and *C*_DHBA_, respectively) were quantified by MCA using AuPRO™ (Pion Inc., Billerica, USA) over wavelength ranges of 240 to 330, 240 to 330 and 260 to 330 nm for CBZ-SAC, CBZ-NIC, and CBZ-DHBA, respectively. All dissolution tests were performed in triplicate.

The residual solids were collected by vacuum filtration. The solid forms of residual particles were identified by powder X-ray diffraction (PXRD). Before PXRD analysis, the collected samples were gently pulverized using a mortar and pestle. A zero-diffraction plate was used as the sample holder. PXRD data were collected from 5 to 35° (2*θ*) (scanning speed: 10° min^-1^; step size: 0.02°; Cu Kα radiation (15 mA, 40 kV)) (MiniFlex, Rigaku Corporation, Tokyo, Japan).

## Results

### UV spectra of carbamazepine and coformers

**Figure 2.**
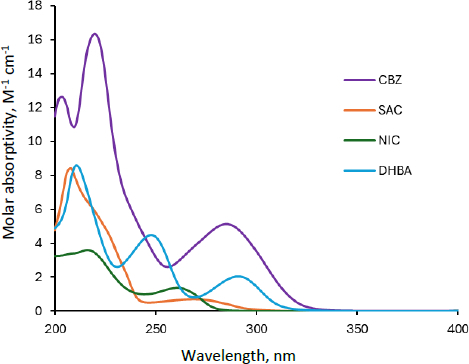
UV spectra of carbamazepine, saccharin, nicotinamide and 2,4-dihydroxybenzoic acid at pH 6.5

The molar absorptivity (*ε*) of CBZ, SAC, NIC, and DHBA is shown in Figure 2. Because these molecules exhibit markedly different UV spectra, MCA can be applied to quantify the components of the cocrystals. In the case of CBZ-SAC and CBZ-NIC, CBZ has been quantified by UV absorbance at 320 nm in previous studies [6] because these coformers exhibit no absorbance above 300 nm. However, because the UV spectra of DHBA extend close to 320 nm, above which the absorbance of CBZ becomes smaller, quantification of CBZ using single-wavelength UV absorbance is more challenging for CBZ-DHBA. Furthermore, the UV spectra of all coformers are interfered with by that of CBZ. In the present study, MCA was successfully applied to quantify each component.

### Dissolution profile of carbamazepine - saccharin

**Figure 3.**
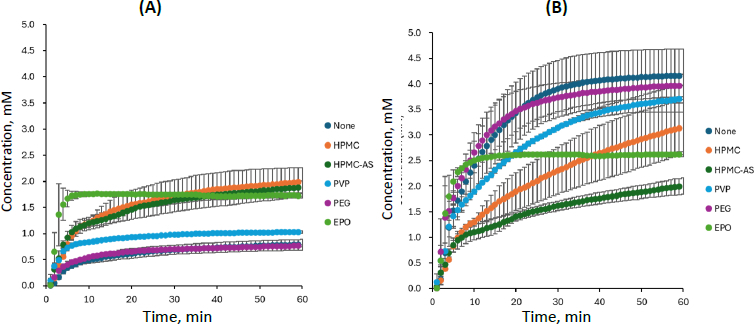
Dissolution profiles of (A) CBZ and (B) SAC from CBZ-SAC, mean ± S.D., *N* = 3

**Figure 4.**
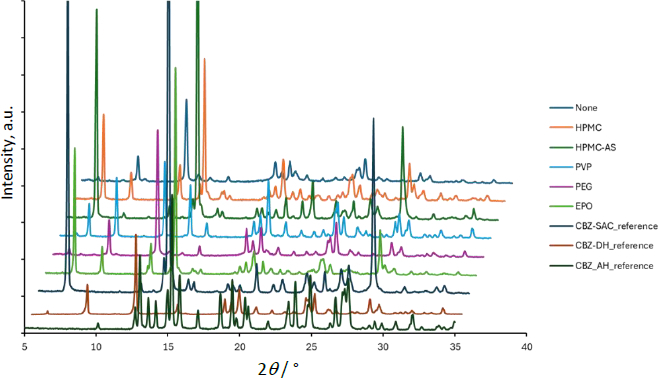
PXRD data of the residual solid after 60 min in the dissolution test of CBZ-SAC. The data without horizontal offset is available in the supplemental information

The dissolution profile of CBZ-SAC is shown in Figure 3, and the PXRD data of the residual solid after 60 min are presented in Figure 4. The dissolution profile of CBZ from CBZ-SAC observed in this study was comparable to the previously reported profiles obtained using the conventional overhead-paddle agitation method and single-wavelength UV absorbance at 320 nm for CBZ quantification (except for EPO, discussed below) [6]. In the present study, the dissolution profiles of CBZ and the coformer (SAC) from CBZ-SAC were measured simultaneously for the first time by applying MCA to *in situ* UV spectra in the RV-μDISS system.

In the absence of a PPI, CBZ was slowly released from CBZ-SAC and dissolved into the medium, reaching a maximum concentration corresponding to only 20 % dissolution (*C_CBZ_* ≈ 0.8 mM). In contrast, SAC was rapidly and completely released from CBZ-SAC (*C_SAC_* ≈ 4.2 mM). Little to no residual PXRD peak of CBZ-SAC was observed after 60 min of dissolution, indicating complete transformation to CBZ dihydrate (CBZ-DH).

In the presence of HPMC, HPMC-AS, and EPO, the dissolution of CBZ from CBZ-SAC was markedly enhanced, whereas that of SAC was reduced compared with the condition without a PPI. The dissolved CBZ concentration reached about 2 mM, which is supersaturated relative to the equilibrium solubility of CBZ-DH (about 1.0 mM). The dissolution behaviour observed in the presence of PVP was intermediate between these two cases. In the case of EPO, CBZ dissolution proceeded most rapidly during the initial phase; however, the process ceased after approximately 5 min. This result differed from a previous report [6], which might be attributable to differences in the agitation method. In the presence of HPMC, HPMC-AS, EPO, and PVP, PXRD peaks corresponding to residual CBZ-SAC were observed after 60 min of dissolution. In contrast, in the case of PEG, little to no effect on the dissolution profiles of either CBZ or SAC was observed, and no PXRD peaks corresponding to residual CBZ-SAC were detected.

### Dissolution profile of carbamazepine - nicotinamide

**Figure 5.**
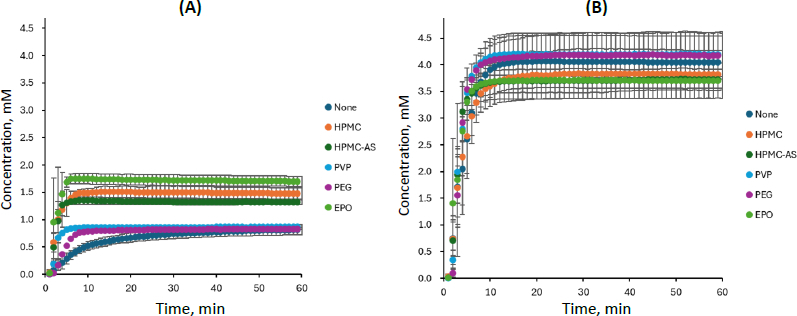
Dissolution profiles of (A) CBZ and (B) NIC from CBZ-NIC, mean ± S.D., *N* = 3

**Figure 6.**
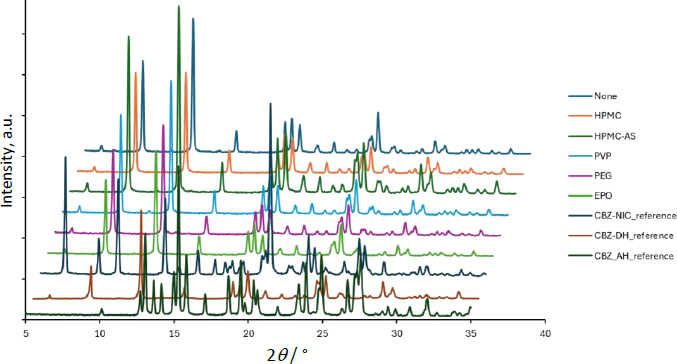
PXRD data of residual solid after 60 min in the dissolution test of CBZ-NIC. The data without horizontal offset is available in the supplemental information

The dissolution profile of CBZ-NIC is shown in Figure 5, and the PXRD data of the residual solid after 60 min are presented in Figure 6. The dissolution profile of CBZ from CBZ-NIC observed in this study was consistent with those previously obtained using a conventional overhead paddle method and single-wavelength UV absorbance [4]. In the present study, the dissolution profile of the coformer (NIC) from CBZ-NIC was measured for the first time. In addition, the effects of PPIs other than HPMC were newly characterized.

Similar to the case of CBZ-SAC, the dissolution of CBZ from CBZ-NIC was enhanced, whereas that of NIC was reduced, by the addition of HPMC, HPMC-AS, and EPO, although the magnitude of this effect was less pronounced than that observed for CBZ-SAC. In all polymer conditions, little to no PXRD peak corresponding to residual CBZ-NIC was observed after 60 min of dissolution.

### Dissolution profile of carbamazepine - 2,4-dihydroxybenzoic acid

**Figure 7.**
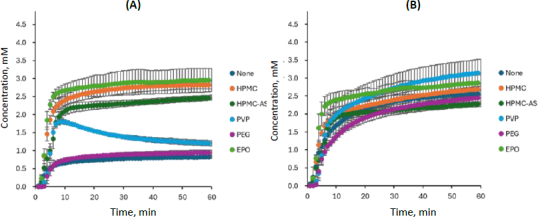
Dissolution profiles of (A) CBZ and (B) DHBA from CBZ-DHBA., mean ± S.D., *N* = 3

**Figure 8.**
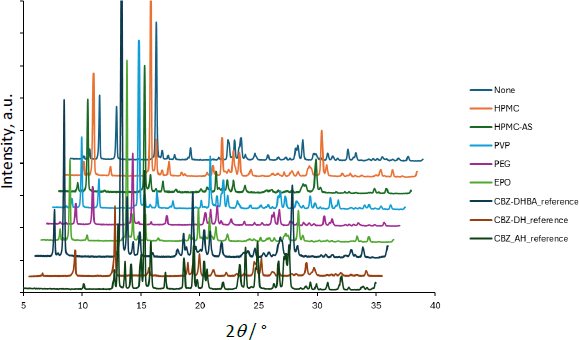
PXRD data of the residual solid after 60 min in the dissolution test of CBZ-DHBA. The data without horizontal offset is available in the supplemental information

The dissolution profiles of CBZ-DHBA are shown in Figure 7, and the PXRD data of the residual solid after 60 min are shown in Figure 8. Similar to CBZ-SAC and CBZ-NIC, PPIs enhanced CBZ dissolution from CBZ-DHBA, except for PEG. However, unlike the observations for CBZ-SAC and CBZ-NIC, the dissolution of DHBA from CBZ-DHBA remained incomplete even in the absence of a PPI. PXRD peaks corresponding to residual CBZ-DHBA were observed after 60 min of dissolution in all conditions. In the PVP solution, the dissolution profiles of CBZ from CBZ-DHBA exhibited a characteristic supersaturation-precipitation behaviour. Although the initial dissolution rate was comparable to that observed with the other polymers (except PEG), PVP was less effective in maintaining the supersaturated concentration of CBZ.

## Discussion

### Comprehensive analysis of dissolution profiles

**Figure 9.**
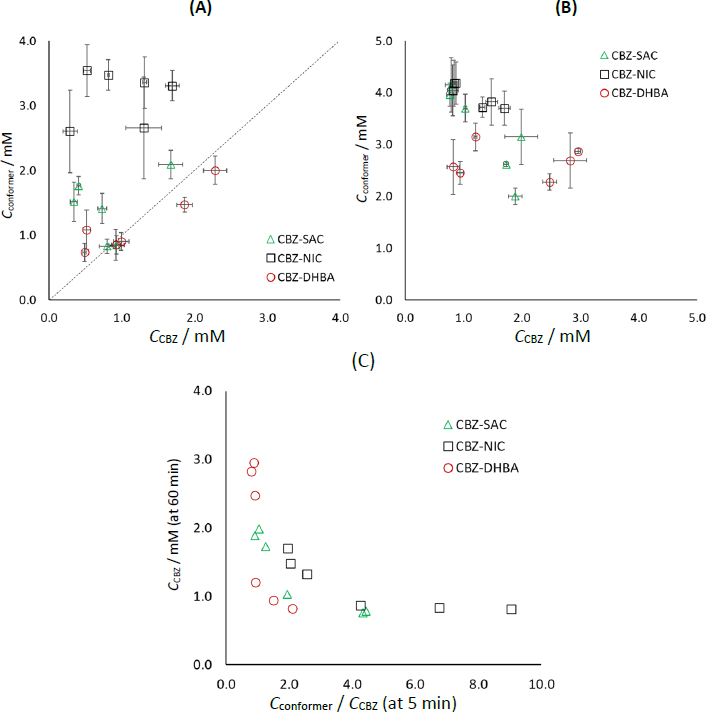
Comparison of *C*_CBZ_ and *C*_coformer_. (A) 5 min, (B) 60 min and (C) *C*_CBZ_/ *C*_coformer_ at 5 min and *C*_CBZ_ at 60 min. The dotted line in (A) corresponds to congruent dissolution. (A) and (B): mean ± S.D., *N* = 3. In (C) *C*_CBZ_/ *C*_coformer_ were calculated from the mean values

Figure 9 presents a comprehensive analysis of the dissolution profiles. At 5 min, CBZ and the coformer tended to dissolve more congruently for CBZ-DHBA and CBZ-SAC compared to CBZ-NIC (Figure 9A). At 60 min, an inverse relationship between *C_CBZ_* and the coformer concentration (*C*_coformer_) was observed for CBZ-SAC and CBZ-NIC, but not for CBZ-DHBA (Figure 9B). When the coformer dissolves more rapidly than CBZ (*C*_coformer_/*C*_CBZ_ > 1 at 5 min), CBZ-DH precipitates on the particle surface more readily, resulting in limited or no supersaturation of CBZ (Figure 9C). In contrast, when CBZ and the coformer dissolve congruently during the initial dissolution phase (*C*_coformer_/*C*_CBZ_ ≈ 1 at 5 min), the supersaturation of CBZ would be enhanced in the later phase. To improve the dissolution performance of CBZ cocrystals, it is important to select an appropriate coformer-PPI combination that promotes the congruent dissolution of CBZ and the coformer.

### Mechanism of cocrystal dissolution and the effect of precipitation inhibitors

Previous cocrystal dissolution studies have suggested that higher coformer solubility tends to result in lower supersaturation in the bulk phase for CBZ [4] and several other drugs [22-24]. When CBZ and a coformer are rapidly released from the cocrystal surface, the local concentration of CBZ at the particle surface increases, thereby accelerating precipitation of CBZ-DH at the particle surface via particle surface solution-mediated phase transformation (PS-SMPT), which consequently leads to lower supersaturation in the bulk phase [25].

In the case of CBZ-SAC and CBZ-NIC, PS-SMPT from the CBZ cocrystals to CBZ DH was previously found to occur rapidly, within seconds (CBZ-NIC > CBZ-SAC), at the particle surface of the cocrystal, thereby suppressing the dissolution of CBZ from the cocrystals [4]. However, PS-SMPT has not been confirmed for CBZ-DHBA. In the present study, in the absence of a PPI, the bulk phase concentration of CBZ released from CBZ-DHBA remained below 2 mM, at which no bulk phase SMPT (BP-SMPT) was observed [25]. Therefore, BP-SMPT can be excluded for CBZ-DHBA, as well as for CBZ-SAC and CBZ-NIC. In the absence of a PPI, it was difficult to distinguish the initial dissolution rate of CBZ-DHBA from those of CBZ-SAC and CBZ-NIC, because the bulk phase CBZ concentration rapidly reached the solubility of CBZ-DH in all cases [4]. However, the water solubility of DHBA (0.038 M at 25 °C; Data retrieved from PubChem) suggests that PS-SMPT in CBZ-DHBA would be less significant than that in CBZ-NIC (4.1 M). Consistent with this, PXRD data indicated that PS-SMPT occurred to a lesser extent in CBZ-DHBA than in CBZ-NIC.

Although the solubility of SAC (0.022 M) is slightly lower than that of DHBA, the release of SAC was faster than that of DHBA in the absence of a PPI. This observation suggests that coformer release is not determined solely by coformer solubility.

**Figure 10.**
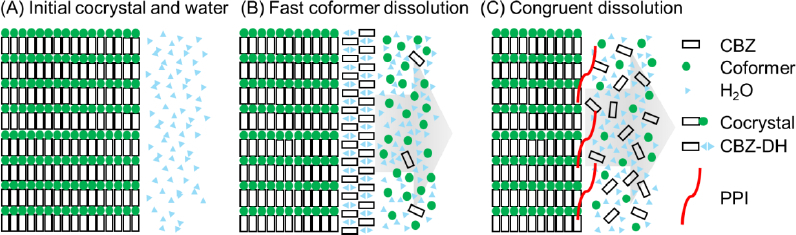
Hypothetical cocrystal dissolution mechanism. When CBZ and the coformer are rapidly released from the cocrystal surface, the local concentration of CBZ at the particle surface would be higher, and the precipitation of CBZ DH would be faster (B). PPIs would reduce the release of CBZ and the coformer (C). This can reduce the local concentration of CBZ, and therefore, slows the PS-SMPT of CBZ-DH at the cocrystal surface. PPIs can also directly inhibit CBZ-DH nucleation and/or crystal growth

Based on the results of this study, a hypothetical mechanism for the effect of PPIs on PS-SMPT is illustrated in Figure 10. Previously, it was assumed that a PPI suppresses PS-SMPT by inhibiting the precipitation (crystal nucleation and/or crystal growth) of CBZ-DH at the particle surface, resulting in an increase in supersaturation in the bulk phase [4]. However, the results of the present study showed that a PPI also reduced the release of the coformers. If a PPI only suppressed the precipitation of CBZ-DH at the cocrystal surface, the release of the coformer would be expected to increase. Therefore, a PPI may also interfere with the molecular dissociation of CBZ and the coformer from the cocrystal surface, resulting in a slower release of both CBZ and the coformer. This effect may reduce the local CBZ concentration at the particle surface and, consequently, slow the precipitation of CBZ-DH. Such an effect may represent an additional mechanism by which polymers suppress rapid PS-SMPT. This mechanism has been hypothesized for CBZ-maleic acid and CBZ-fumaric acid monohydrate cocrystals [5], and the results of the present study supported this mechanism. This mechanism may also explain the different effects of a PPI on PS-SMPT and BP-SMPT [6]. In the case of CBZ-DHBA, PVP and the other PPIs exhibit comparable effectiveness in increasing the initial dissolution rate, whereas PVP was less effective than the other PPIs in inhibiting BP-SMPT of CBZ-DH [6]. This discrepancy further suggests that the mechanisms underlying PS-SMPT and BP-SMPT may differ [4-6].

### Limitations of this study

The standard deviation in the dissolution profiles (Figures 3, 5, and 7) differed among each cocrystal and polymer. The reason for this is not clear. Since the data variation tended to be more pronounced in the initial dissolution phase, it may be related to the wettability of each coformer and polymer solution.

## Conclusions

In conclusion, the dissolution profiles of CBZ and the coformers from the cocrystals were simultaneously quantified by applying MCA to *in situ* UV spectra. The addition of a PPI increased the release of CBZ from a cocrystal, while decreasing the release of the coformer, resulting in more congruent release of CBZ and the coformer. The slower dissociation of the coformer and CBZ from the cocrystal surface may have reduced the local concentration of CBZ, and therefore, slowed the precipitation of CBZ-DH. The congruent dissolution of CBZ and the coformer from the cocrystal was found to be important for inducing supersaturation of CBZ in the bulk phase. This analytical approach provides valuable insights into the dissolution mechanisms of cocrystals and offers a promising tool for coformer and PPI selection for formulation development.

## Supplementary material

Multicomponent regression analysis in AuPRO 6.0 and later, and powder X-ray diffraction data without horizontal offset are available at *https://pub.iapchem.org/ojs/index.php/admet/article/view/3200*, or from the corresponding author on request.


