# Supplementary material for: Simultaneous quantification of drug and coformer during cocrystal dissolution using in situ UV spectroscopy and multicomponent analysis
Source: ADMET DMPK. 2026 Apr 17;14:3274. doi: 10.5599/admet.3274 (PMC13147517; doi:10.5599/admet.3274)
Supplement: Supplementary file 1 [file ADMET-14-3274-S1.pdf]

ADMET & DMPK **14** (2026) S3274

Open Access : ISSN : 1848-7718
http://www.pub.iapchem.org/ojs/index.php/admet

Supplementary material to

Simultaneous quantification of drug and coformer during cocrystal dissolution using *in situ* UV spectroscopy and multicomponent analysis

Shiori Ishida[1], Samuel Lee[2], Balint Sinko[2], Karl Box[2] and Kiyohiko Sugano[1,*]

[1]*Molecular Pharmaceutics Lab., College of Pharmaceutical Sciences, Ritsumeikan University, 1-1-1, Noji-higashi, Kusatsu, Shiga 525-8577, Japan*
[2]*Pion Inc. (UK) Ltd. Forest Row Business Park, Station Road, East Sussex, RH18 5DW, United Kingdom*

ADMET & DMPK **14** (2026) 3247; https://doi.org/10.5599/admet.3274

Multicomponent regression analysis in AuPRO 6.0 and later

Multicomponent regression analysis operates on the assumption that a mixture composed of N components has the total absorption of the mixture at a particular wavelength being the sum of all the absorbance contributions of each component. This assumption holds if the UV signal is not saturated and there is no interaction between the components that influence the absorbance.

This assumption can be represented using Equation (S1):

$$A_{\text{Total}}(\lambda) = \sum_{i-1}^{N} A_{\text{i}}(\lambda) \tag{S1}$$

where $A_i(\lambda)$ is the absorbance of one component of the mixture at a specific wavelength and $A_{\text{Total}}(\lambda)$ is the sum of the absorbance of all components of the mixture at a specific wavelength. The equation can also be written such that the absorbance of one component at a specific wavelength is expressed as the measured absorbance of a standard solution of the component, multiplied by a coefficient, to give Equation (S2).

$$A_i(\lambda) = x_i\, A_{\text{st,i}}(\lambda) \tag{S2}$$

where $A_{\text{st,i}}$ is the measured absorbance of a standard of known concentration of one component. Nd x_i is a coefficient.

Equation (S2) can then be substituted into Equation (S1) to give Equation (S3):

$$A_{\text{Total}}(\lambda) = \sum_{i-1}^{N} x_{\text{i}} A_{\text{st,i}}(\lambda) \tag{S3}$$

This results in a system of N equations and thus N coefficients, which is resolved iteratively to obtain values of x_i for each component, until

$$A_{\text{Total}}(\lambda) - \sum_{i-1}^{N} x_{\text{i}} A_{\text{st,i}}(\lambda) = 0\,,$$

or as close to zero as possible for each wavelength, and then across the whole wavelength range in use as *per* Equation (S4).

$$\chi^2 = \sum_{\lambda_1}^{\lambda_2} \left[A_{\text{Measured}}(\lambda) - \sum_{i-1}^{N} x_{\text{i}} A_{\text{st,i}}(\lambda) \right]^2 \tag{S4}$$

When the values of x_i are found for each component, the calculation of the concentration of each component can be done using Equation (S5):

$$c_i = x_i c_{st} \quad \text{(S5)}$$

where c_{st} is the real concentration of the standard solution whose measured absorbance value was used to determine x_i using Equation (S3).

Powder X-ray diffraction data without horizontal offset

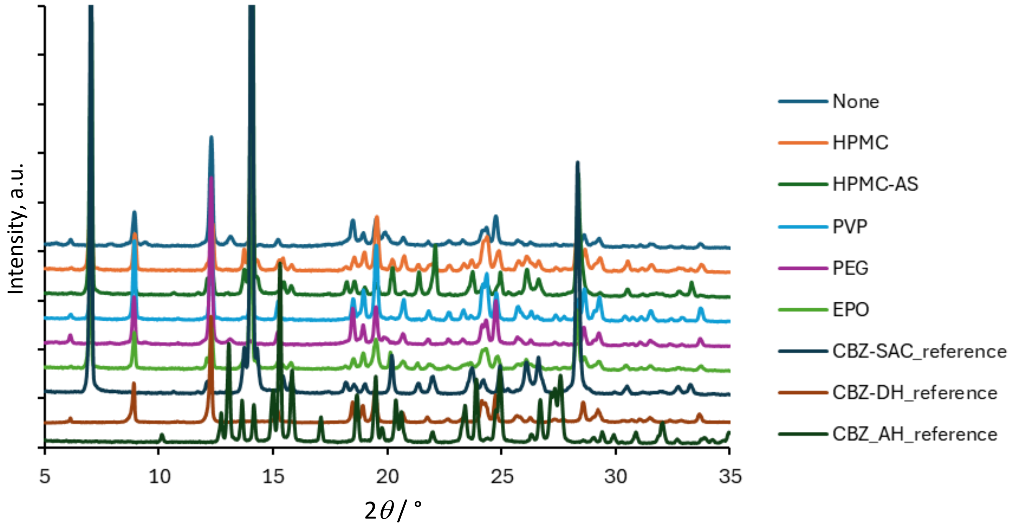

Figure S1. PXRD data of residual solid after 60 min in the dissolution test of CBZ-SAC

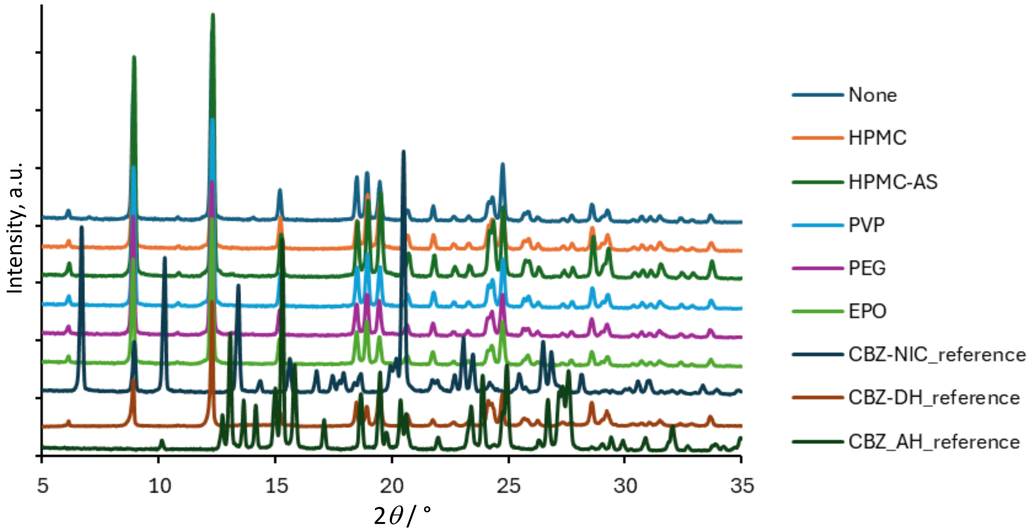

Figure S2. PXRD data of residual solid after 60 min in the dissolution test of CBZ-NIC

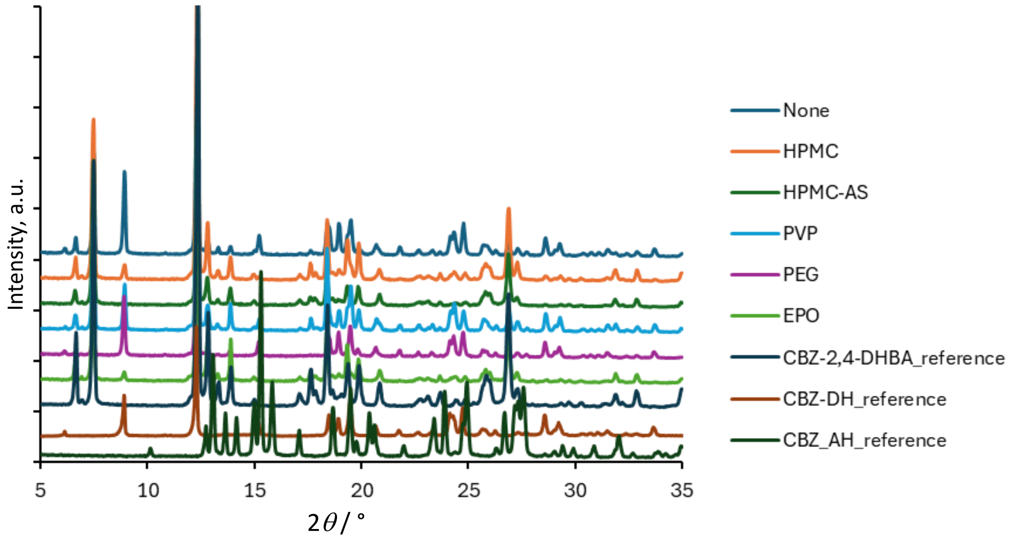

Figure S3. PXRD data of residual solid after 60 min in the dissolution test of CBZ-DHBA

(cc) BY
